# Supplementary material for: Discovery and application of insertion-deletion (INDEL) polymorphisms for QTL mapping of early life-history traits in Atlantic salmon
Source: BMC Genomics. 2010 Mar 8;11:156. doi: 10.1186/1471-2164-11-156 (PMC2838853; doi:10.1186/1471-2164-11-156)
Supplement: Additional file 2 — Information on developed 76 locus single-run INDEL panel in Atlantic salmon. Information on fluorescence labeling, primer concentrations, PCR pooling and links to alignments, INDEL motifs and GENESCAN (Burge and Karlin 1997) predictions of genes/exons are available in html format. [file 1471-2164-11-156-S2.ZIP › Additionalfile2/snpsummary775.html]

```
Cluster 90 Contig 1

prev  Summary    Contig List  next
```

Size of Consensus sequence = 657

Number of sequences = 4

Minimum redundancy = 1

Key

A gi|117447441|gb|EG779660.1|EG779660 EST\_ssal\_evd\_36477 ssalevd thymus Salmo salar cDNA Salmo salar cDNA clone ssal\_evd\_548\_265\_rev 5', mRNA sequence  
B gi|117469429|gb|EG801648.1|EG801648 EST\_ssal\_evd\_58266 ssalevd thymus Salmo salar cDNA Salmo salar cDNA clone ssal\_evd\_578\_241\_fwd 3', mRNA sequence  
C gi|117469430|gb|EG801649.1|EG801649 EST\_ssal\_evd\_58267 ssalevd thymus Salmo salar cDNA Salmo salar cDNA clone ssal\_evd\_578\_241\_rev 5', mRNA sequence  
D gi|117447442|gb|EG779661.1|EG779661 EST\_ssal\_evd\_36478 ssalevd thymus Salmo salar cDNA Salmo salar cDNA clone ssal\_evd\_548\_265\_fwd 3', mRNA sequence

9 SNPs detected

A B C D  cosegregation weighted

119 A A C A   1/9 11.11
174 A - - A   6/9 66.67
175 G - - G   6/9 66.67
176 G - - G   6/9 66.67
177 C - - C   6/9 66.67
178 T - - T   6/9 66.67
449 A G G G   2/9 22.22
503 A G G G   2/9 22.22
560 . C C A   6/9 50.00
